# Peruna and the “Bracers”

**Published:** 1905-12

**Authors:** 


### EDITORIAL NOTES AND COMMENTS.

The Business office of THE JOURNAL-RECORD is No. 1007-1008 Century Building.

The Editorial office is 318-319-320 The Prudential.

Address all Business communications to Dr. M. B. Hutchins, Mgr.

Make remittances payable to THE ATLANTA JOURNAL-RECORD OF MEDICINE.

On matters pertaining to the Editorial and Original Communications address Dr Bernard Wolff, Atlanta.

Reprints of original articles will be furnished at cost price. Requests for the same should always be made *on the manuscript.*

We will present, post-paid, on request, to each contributor of an original article, twenty (20) marked copies of THE JOURNAL-RECORD containing such article.

### PERUNA AND THE "BRACERS."

THE facts brought out by Samuel Hopkins Adams in *Collier's* for October 28th are of such importance and directed against such a flagrant evil that we desire to present a few of the points and do our share in giving publicity to this commendable work.

Peruna is shown to contain 28 per cent. of alcohol, Paine's Celery Compound 21 per cent. and Hostetter's Stomach Bitters 44.3 per cent. These are compared with whiskey, champagne, claret and beer, and the reason for their popularity becomes immediately evident. It is asserted by Dr. A. P. Grinnell, of New York City, that more alcohol is consumed in this country in patent medicines than is dispensed in a legal way by licensed vendors, barring the sale of ales and beer.

Peruna is not claimed to be "a cure-all," being chiefly of value in the so-called "catarrhal diseases," such as pneumonia and tuberculosis, which are merely "catarrh of the lungs," dyspepsia, catarrh of the stomach; enteritis, catarrh of the intestines; appendicitis, catarrh of the appendix; Bright's disease, catarrh of the kidneys, etc. Other diseases not placed in this class but yielding to Peruna (?) are colic, mumps, neuralgia, yellow fever, women's complaints, rheumatism and convulsions. "That alcohol and water, with a little coloring matter and one-half of one per cent. of mild drugs, will cure all or any of the ills listed above, is too ridiculous to need refutation."

A considerable proportion of tubercular patients show a history of having taken the Peruna type of medicines, early in the disease, diminishing the resisting power and delaying the proper treatment. "It is not as a fraud upon the sick alone that Peruna is baneful, but as the maker of drunkards also." The Government has forbidden the sale of it to the Indians.

An interesting letter from Wyoming is quoted in which two men suffering from delirium tremens, and one dead, were given as the result of Peruna taken on a camping trip through Yellowstone Park.

Further information is brought out as to testimonials and the methods of obtaining them. The "clergymen who endorse Duffy's pure malt whiskey" were followed up and were found to be a Deputy Internal Revenue Collector, a proprietor of a get-married-quick matrimonial bureau, and the third, Mr. McLeod, was called to trial by his presbytery for giving this endorsement and was allowed to "resign."

"Warner's Safe Cure, together with all the Warner remedies, is leased, managed and controlled by the New York and Kentucky Distilling Company, manufacturers of standard whiskeys which do not pretend to remedy anything but thirst."

